# Therapeutic inhibition of mitochondrial function induces cell death in starvation-resistant renal cell carcinomas

**DOI:** 10.1038/srep25669

**Published:** 2016-05-09

**Authors:** Takahiro Isono, Tokuhiro Chano, Junji Yonese, Takeshi Yuasa

**Affiliations:** 1 Central Reseach Laboratory, Shiga University of Medical Science, Tsukinowa-cho, Seta, Otsu, Shiga 520-2192, Japan; 2 Departments of Clinical Laboratory Medicine, Shiga University of Medical Science, Otsu, Shiga 520-2192, Japan; 3 Departments of Urology, Cancer Institute Hospital, Japanese Foundation for Cancer Research, Tokyo 135-8550, Japan.

## Abstract

Renal cell carcinomas (RCC) have two types of cells for carbon metabolism and for cell signaling under nutrient-deprivation conditions, namely starvation-resistant and starvation-sensitive cells. Here, we evaluated the mitochondrial characteristics of these cell types and found that the resistant type possessed higher activities for both mitochondrial oxidative phosphorylation and glycolysis than the sensitive types. These higher activities were supported by the stored carbon, lipid and carbohydrate sources, and by a low level of mitochondrial reactive oxygen species (ROS) due to sustained SOD2 expression in the resistant RCC cells. In metastatic RCC cases, higher SOD2 expression was associated with a significantly shorter survival period. We found that treatment with the drugs etomoxir and buformin significantly reduce**d** mitochondrial oxidative phosphorylation and induced cell death under glucose-deprivation conditions in starvation-resistant RCC cells. Our data suggest that inhibitory targeting of mitochondria might offer an effective therapeutic option for metastatic RCC that is resistant to current treatments.

Renal cell carcinoma (RCC) is the urological malignancy with the highest rate of mortality and is currently increasing in incidence^1^. Radical nephrectomy remains the standard and only curative therapy for patients with localized RCC. However, after initial diagnosis, one third of RCC patients exhibit visceral metastases and up to half of the remainder eventually develop distant metastases^2^. Currently, molecular targeting therapies employing two major subgroups of agents are used for patients with metastatic RCC: tyrosine kinase inhibitors, including Sorafenib (Nexavar, Bayer), Sunitinib (Sutent, Pfizer), Pazopanib (Votrient, GlaxoSmithKline), and Axitinib (Inlyta, Pfizer)^3^,^4^,^5^,^6^,^7^; and specific inhibitors of the mammalian target of rapamycin (mTOR) kinase, Temsirolimus (Torisel, Pfizer) and Everolimus (Afinitor, Novartis)^8^,^9^. The common rationale for use of these agents to suppress cancer development is based on nutrient-deprivation, including inhibition of tumor angiogenesis, rather than direct inhibition of the cancer cells. Glucose is the major nutrient denied to cells following inhibition of angiogenesis.

In a previous study, we demonstrated that there were two types of cells in RCC for carbon metabolism and for cell signaling under glucose deprivation^10^, and suggested that differences between these cell types might be a key factor in the efficacy of novel targeting therapies. One type of cancer cells, which we termed “starvation-sensitive”, produced *N*-linked (β-*N*-acetylglucosamine)_2_ [*N*-GlcNAc_2_]-modified glycoproteins under glucose deprivation^11^. These glycoproteins promoted an unfolded protein response (UPR) in the endoplasmic reticulum and the cells were induced to arrest at G2/M causing a mitotic “catastrophe”^12^, and death of the starvation-sensitive RCC cells^10^. The other type of cell, termed “starvation-resistant”, did not show *N*-GlcNAc_2_-modified protein accumulation or cell death; this behavior suggested they were dormant-state cells under glucose deprivation^10^. It was suggested that further investigations would clarify the differences in these cell types and help to develop a more therapeutic approach.

Here, we analyzed respiration and glycolysis in RCC using a Flux analyzer, that measures the oxygen consumption rate (OCR) and extracellular acidification rate (ECAR)^13^. Our analysis demonstrated an increased level of respiration in starvation-resistant cells compared to sensitive cells that associated with a poorer prognosis. Additionally, we found that targeting the mitochondria could offer an effective therapeutic option for RCC.

## Results

### Starvation-resistant RCC cell lines possess high mitochondrial oxidative phosphorylation activity and abundant glycolytic reserves

**Figure 1:**
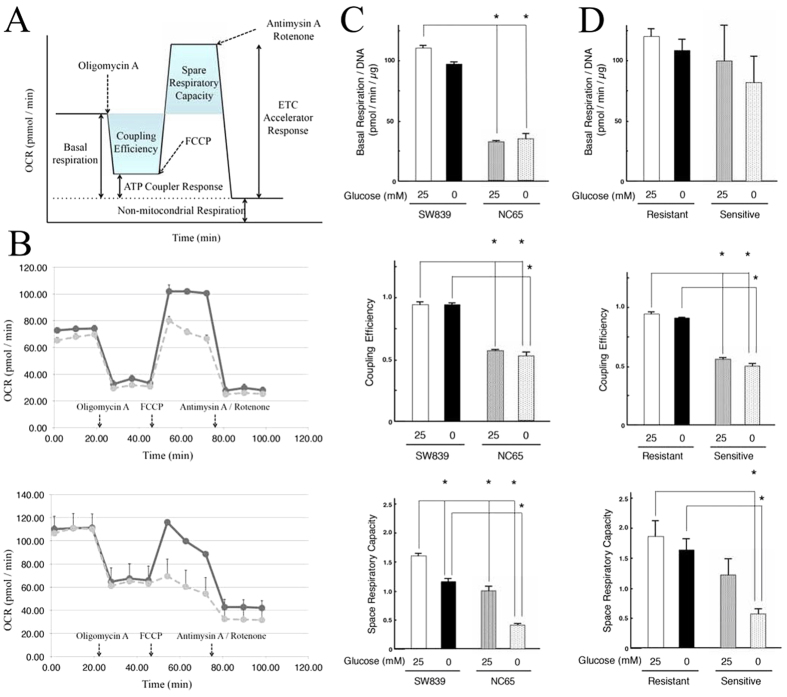
Mitochondrial oxidative phosphorylation activity in starvation-resistant and -sensitive RCC cells. **(A**) OCR (oxygen consumption rate) profile. See text regarding spare respiratory capacity and coupling efficiency. (**B**) Kinetic OCR responses of starvation-resistant (SW839, upper) and -sensitive (NC65, bottom) cells. Both cell types were cultured with (solid line) or without glucose (dotted line). (**C,D**) Basal respiration (upper), coupling efficiency (middle) and spare respiratory capacity (bottom), were calculated for SW839 and NC65 cells (**C**), and for both groups of starvation-resistant and -sensitive cell lines (**D**), were calculated. Basal respiration was normalized against DNA content, which reflected cell number. Error bars represent standard errors from 3–5 independent experiments. Asterisks (*) indicate statistically significant differences (p < 0.05) between both cells/groups. Note that there were clear differences between SW839 and NC65 cells for spare respiratory capacity, which was significantly decreased in NC65 cells under glucose-deprived conditions. NC65 cells showed significantly lower basal respiration and coupling eficiency than SW839 cells, but there were no clear changes between cultures with (25 mM) or without (0 mM) glucose.

We investigated mitochondrial oxidative phosphorylation in RCC cell lines using XF Cell Mito Stress Test Kit and found that starvation-resistant cells possessed significantly higher spare respiratory capacities than the sensitive cells under glucose-deprivation condition (Fig. 1 and Supplementary Table S1). However, there were no significant differences in basal respiration between the two types of cells in culture medium with 25 mm glucose or without glucose (0 mM) glucose. The coupling efficiency of stavation-resistant cells (~90%) was significantly higher than that of sensitive cells (~60%) in culture medium with or without glucose. There was no correlation between the three metrics (basal respiration, spare respiratory capacities and coupling efficiency) and mitochondrial numbers in each cell (Supplementary Table S2). These results suggested that starvation-resistant cells possessed higher mitochondrial oxidative phosphorylation activities than sensitive cells.

**Figure 2:**
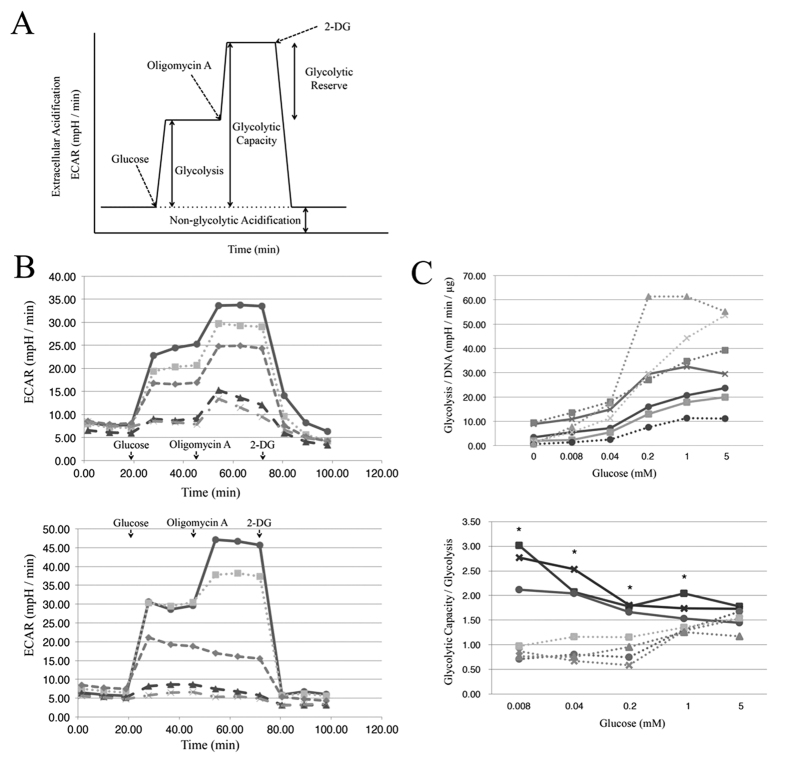
Glycolysis activity in RCC. (**A**) ECAR (extracelluar acidification rate) profile. (**B**) Kinetic ECAR responses in starvation-resistant (SW839, upper) and -sensitive (NC65, bottom) cells in cultures with different concentrations of glucose: circles and solid line, 5 mM; squares and dotted line, 1 mM; diamonds and short dashed line, 0.2 mM; triangles and long dashed line, 0.04 mM; and crosses and interrupted line, 0.008 mM.(**C**) Glycolysis (upper) and glycolytic reserve (bottom), shown as glycolytic capacity/glycolysis. Glycolysis was normalized against DNA content, which reflects cell numbers. Data show the averages of two independent experiments. Circles and solid line, SW839 cells; squares and solid line, VMCR-RCW; crosses and solid line, KMRC-1; circles and dotted line, NC65; squares and dotted line, ACHN; triangles and dotted line, Caki1; and crosses and dotted line, Caki2 cells. Asterisks (*) indicate statistically significant differences (p < 0.05) between both groups of resistant and sensitive cell lines. Note that there were no clear differences between the two types of RCC cells in glycolytic activities. However, there were clear differences for glycolytic capacity. All 3 resistant cell lines (SW839, VMCR-RCW, and KMRC-1) possessed high glycolytic reserves under low (0.008–1 mM) glucose conditions, and similarly at high (5 mM) concentrations; however, there was no glycolytic reserve in the sensitive cell lines (NC65, ACHN, Caki1 and Caki2).

Next, we used the XF Glycolysis Stress Test Kit to compare the glycolytic reserves and activities in the two cell types under different glucose concentrations. Kinetic ECAR responses, which measure glycolysis rates, in starvation-resistant (SW839, Fig. 2B upper) and starvation-sensitive (NC65, Fig. 2B bottom) cells showed that SW839 could maintain glycolytic capacities and reserves even in cultures with lower glucose concentrations (0.2 - 0.008 mM) compared with those of NC65. Among all 7 cell lines tested in the present study, the three starvation-resistant cell lines showed significantly higher values for glycolytic capacity/glycolysis under lower (1–0.008 mM) glucose concentrations, as compared with the four starvation-sensitive cell lines (Fig. 2C bottom). Glycolytic activities increased with glucose concentrations, and there were no significant differences between the two types of cells even at concentrations of 5–0.008 mM glucose (Fig. 2C upper). Thus, the carbon source for respiration in starvation-resistant cells could be supplied from the glycolytic reserve under glucose-deprived environments.

Together, the two assays for mitochondrial oxidative phosphorylation and for glycolysis demonstrated that starvation-resistant RCC cell lines possessed especially better mitochondrial characteristics and had abundant carbon stores compared to starvation-sensitive cells.

### Lipid and hydrocarbon reserves and less mitochondrial ROS in starvation-resistant RCC cell lines

**Figure 3:**
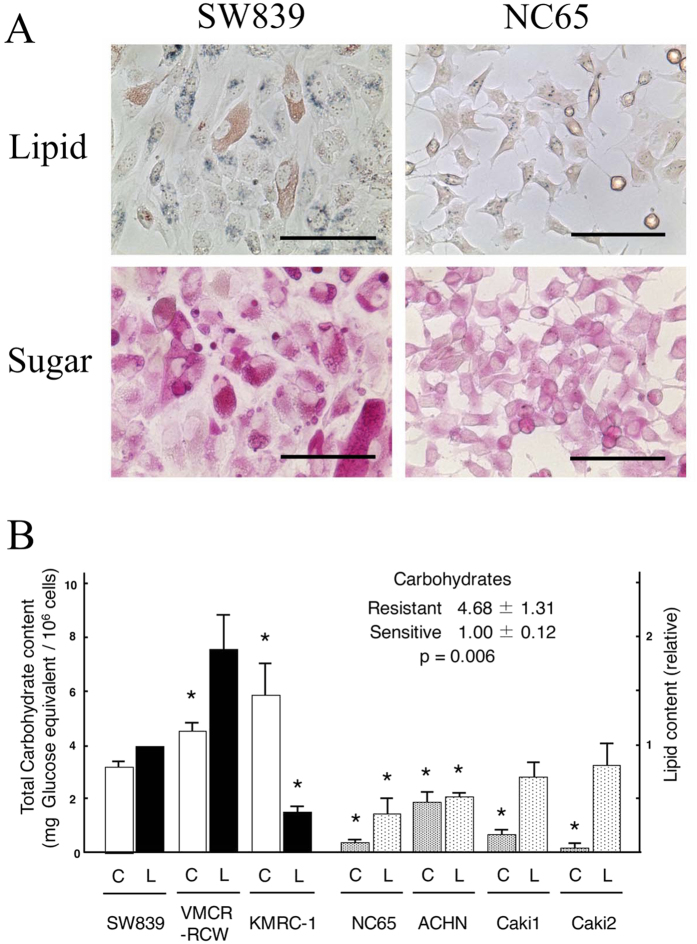
The stock of lipid and hydrocarbon stores in RCC cell lines. (**A**) Lipid (Oil Red O, upper) and sugar (PAS, lower) stainings of starvation-resistant (SW839, left) and -sensitive (NC65, right) cells. The scale bars correspond to 100 μm.(**B**) Hydrocarbon and lipid contents of RCC cells by colorimetric assays. Asterisks (*) indicate statistically significant differences (p < 0.05) in respect of the values of SW839 cells. Note that the hydrocarbon (C) and lipid (L) contents in starvation-resistant cell lines (SW839, VMCR-RCW, and KMRC-1) were considerably higher than in the sensitive cell lines (NC65, ACHN, Caki1 and Caki2).

We used histochemical staining and colorimetric methods to evaluate lipid and hydrocarbon contents in the two cell types (Fig. 3). Oil Red O and PAS staining showed that the starvation-resistant cell line SW839 contained more lipids and sugars than the sensitive cell line NC65. In the colorimetric assay of all 7 cell lines, starvation-resistant cells showed significantly higher hydrocarbon contents than starvation-sensitive cells. The colorimetric assay of lipid contents showed no consistent differences but considerable variation between the two types of cells. The starvation-resistant cell line, KMRC-1, had the highest hydrocarbon content but an exceptionally low lipid content. In this cell line, the high hydrocarbon content could support enough higher stores of carbon. This analysis demonstrated that the increase in mitochondrial oxidative phosphorylation and glycolysis in starvation-resistant cells were supported by higher stores of carbon.

**Figure 4:**
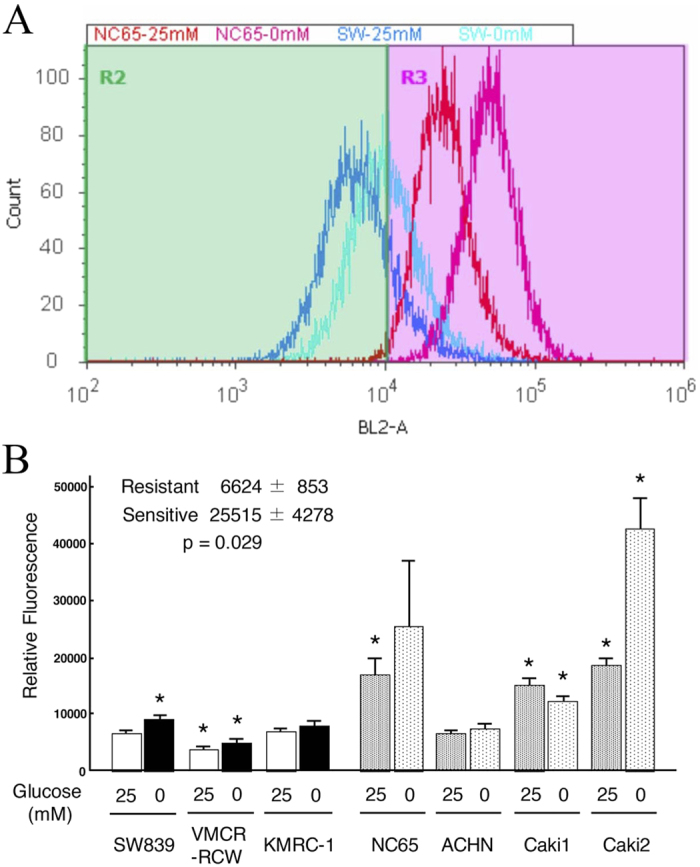
Flow cytometric analysis of mitochondrial ROS in RCC cell lines. (**A**)Mitochondrial ROS were quantified by MitoSOXRed in starvation-resistant (SW839, blues) and -sensitive (NC65, reds) cells. SW839 and NC65 cells were cultured in medium containing either 25 mM or 0 mM glucose. The key to the treatment groups is given on the graph. (**B**) Relative fluorescence scores of the different cell lines. Asterisks (*) indicates statistically significant differences (p < 0.05) in respect of the values of SW839 cells with 25 mM glucose. Note that mitochondrial ROS were significantly lower in the starvation-resistant cell lines (SW839, VMCR-RCW, and KMRC-1) with or without glucose, and higher in the sensitive cell lines (NC65, Caki1 and Caki2). ACHN, a sensitive cell line, had similar levels of mitochondrial ROS to those of SW839 cell line.

**Figure 5:**
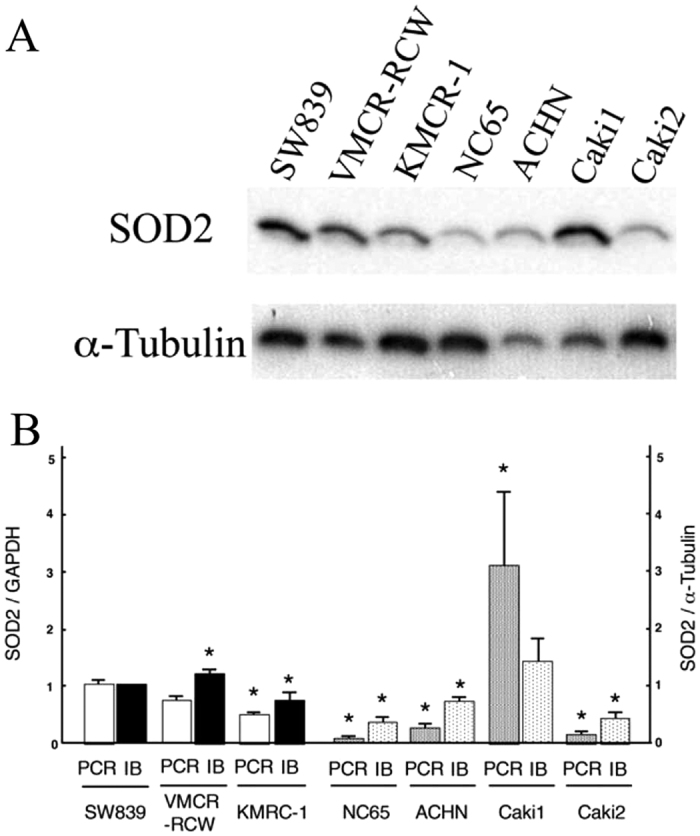
Expression of SOD2 in RCC cell lines. (**A**) Immunoblots for SOD2 and α-tubulin. (**B**) Quantitative RT-PCR (PCR, left column) and immunoblots (IB, right column). The cells were incubated in medium containing 25 mM glucose. Transcriptional and protein expressions were normalized against *GAPDH* and α-tubulin, respectively. Error bars represent standard errors from three independently replicate experiments. Asterisks (*) indicate statistically significant differences (p < 0.05) in respect of the values of SW839 cells. Note that SOD2 expression was higher both for mRNA and protein in the starvation-resistant cell lines (SW839, VMCR-RCW, and KMRC-1) than the sensitive cell lines (NC65, ACHN and Caki2). The sensitive cell line Caki1 had exceptionally high SOD2 expression.

We used MitoSOXRed to investigate whether mitochondrial reactive oxygen species (ROS) contributed to the high coupling efficiency of mitochondrial oxidative phosphorylation in starvation-resistant cells (Fig. 4). A low level of mitochondrial ROS was present in starvation-resistant cells compared to sensitive cells, with or without glucose. The starvation-sensitive cell line, ACHN, was exceptional in showing no significant difference compared to the starvation-resistant cell line, SW839. We also screened the cells to determine whether mitochondrial manganese-dependent superoxide dismutase (SOD2) contributed to the lower mitochondrial ROS in starvation-resistant cells. In both immunoblots and qPCR assays, SOD2 was abundantly expressed at both protein and mRNA levels, especially in the starvation-resistant cells compared to sensitive cells. However, SOD2 expression was also exceptionally high in the sensitive cell line, Caki1 (Fig. 5). These analyses showed that both lower levels of mitochondrial ROS and higher levels of SOD2 expression were characteristic of all starvation-resistant cell lines, although each starvation-sensitive cell line, ACHN or Caki1, showed unilaterally a lower level of mitochondrial ROS or a higher level of SOD2 expression, respectively. Our findings indicate that higher levels of SOD2 expression contributes, in part at least, to differences in the high coupling efficiency of mitochondrial oxidative phosphorylation through a reduction of mitochondrial ROS in starvation-resistant cells.

### Higher SOD2 expression level predicts a poorer prognosis in metastatic RCC patients

**Figure 6:**
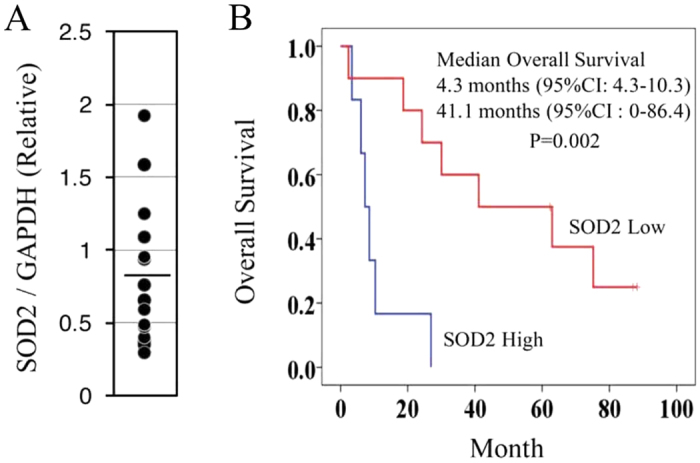
SOD2 expression level predicts prognosis for metastatic RCC patients. (**A**) Samples from patients with clinically metastatic RCC were used to determine *SOD2* expression level using qRT-PCR and were normalized against *GAPDH*. The solid line indicates the average level of *SOD2* expression. **B,** Kaplan-Meier survival curves for SOD2 in metastatic RCC patients. The RCC patients were divided into high or low *SOD2* expression groups. The high *SOD2* expression group showed significantly shorter survival periods than the low expression group after targeting chemotherapies (Log-rank test, p = 0.002).

We investigated whether SOD2 expression level might be a predictor of the clinical outcome in metastatic RCC patients treated with inhibitory agents against tyrosine kinase and mTOR. For this purpose, the characteristics and demographic data of 16 patients were considered (Supplementary Table S3). These patients could be separated into two groups based on average SOD2 expression level in the primary lesions (Fig. 6A); cases with higher SOD2 expression were found to have significantly shorter survival periods (4.3 months) than patients with lower SOD2 expression (41.1 months) (Fig. 6B). Importantly, the comparison indicated that the patients with higher SOD2 expression level were resistant to the drugs used here, probably due to a higher level of mitochondrial oxidative phosphorylation in starvation-resistant cells, and this characteristic resulted in a significantly poorer prognosis.

### Therapeutic inhibition of mitochondrial function induces cell death in starvation-resistant cells

**Figure 7:**
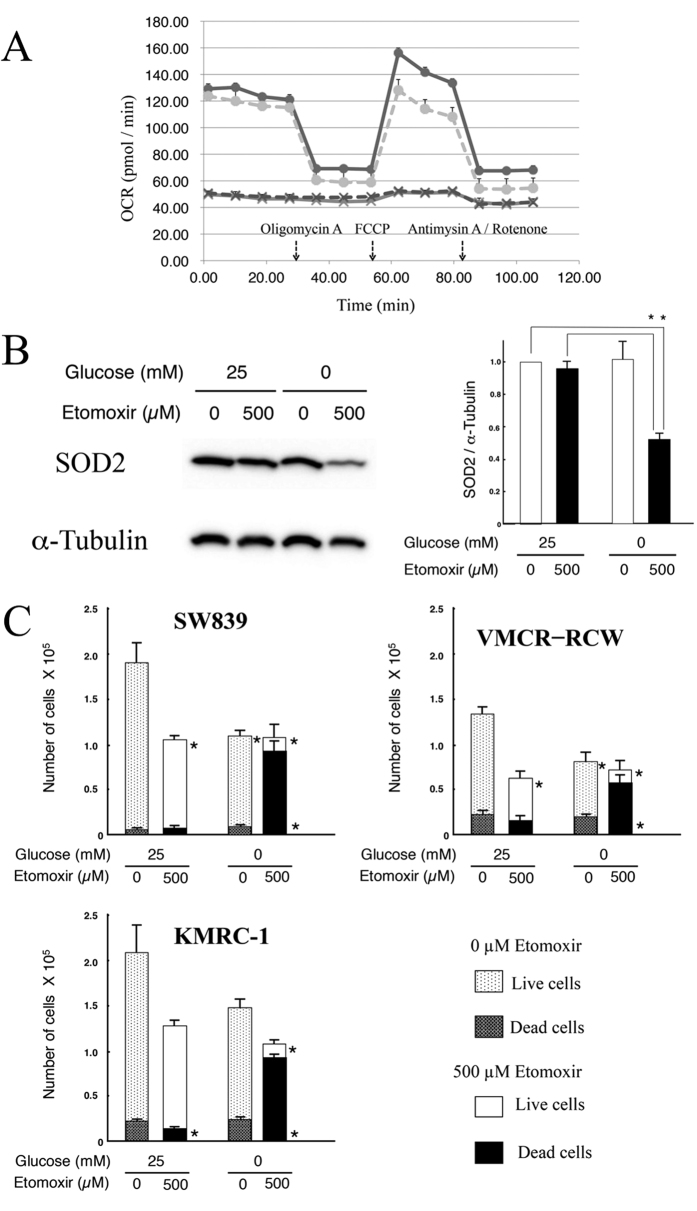
Etomoxir inhibits mitochondrial function and induces cell death in starvation-resistant RCC cell lines under glucose depriviation. (**A**) Kinetic OCR response of SW839 cells. The treatment groups were: 25 mM glucose, circles and solid line; 0 mM glucose, circles and broken line; 25 mM glucose and 500 μM etomoxir, crosses and solid line; and 0 mM glucose and 500 μM etomoxir, crosses and broken line. (**B**) Immunoblot analysis of SOD2. SOD2 expression was normalized against α-tubulin, as the control for the untreated (0 μM etomoxir) cells in high (25 mM) glucose medium. Left panel, immunoblots for SOD2 and α-tubulin; right panel, quantitative analysis of SOD2. Asterisks (*) indicate statistically significant differences (p < 0.05) in respect of the values of SW839 cells under 25 mM glucose with or without etomoxir.(**C**) Starvation-resistant cell lines, SW839, VMCR-RCW, and KMRC-1, were cultured in 25 mM or 0 mM glucose medium with or without 500 μM etomoxir for 24 h. The numbers of living and dead cells were counted using the trypan-blue exclusion assay. Error bars represent standard errors from three replicate experiments. Asterisks (*) indicate statistically significant differences (p < 0.05) in respect of the numbers of living (upper) or dead cells (lower) under control conditions (25 mM glucose without etomoxir). Note that etomoxir inhibited the function of mitochondrial oxidative phosphorylation (**A**) and the expression of SOD2 protein (**B**) in SW839 cells, and induced cell death (**C**) in all starvation-resistant cell lines (SW839, VMCR-RCW and KMRC-1) under glucose depriviation.

To identify possible therapeutic options, inhibitors that target mitochondrial oxidative phosphorylation in starvation-resistant cells were trialed. Etomoxir (500 μM), an inhibitor of beta-oxidation from fatty acids, inhibited mitochondrial oxidative phosphorylation (Fig. 7A) and SOD2 expression, and induced a significantly greater rate of cell death in SW839 cells and the other resistant cell lines under glucose deprivation conditions (Fig. 7B,C, and Supplementary Table S4).

**Figure 8:**
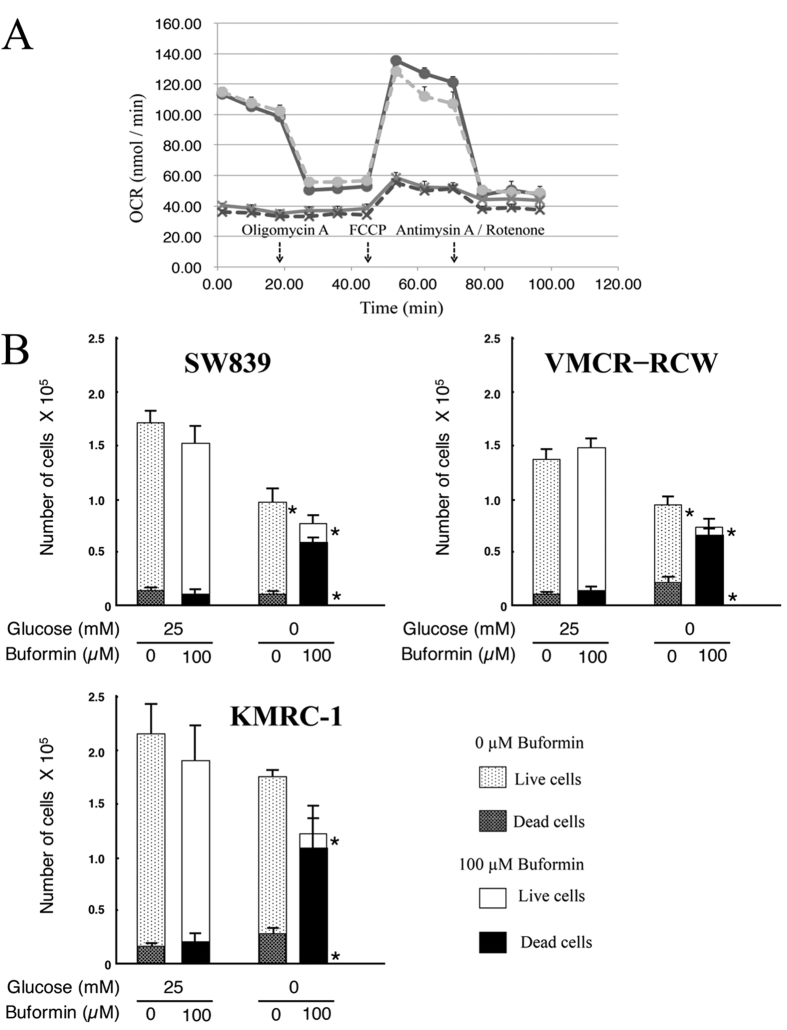
Buformin inhibits mitochondrial function and induces cell death in starvation-resistant RCC cell lines under glucose depriviation. (**A**) Kinetic OCR response of SW839 cells. The treatment groups were: 25 mM glucose, circles and solid line; 0 mM glucose, circles and broken line; 25 mM glucose and 100 μM buformin, crosses and solid line; and 0 mM glucose and 100 μM buformin, crosses and broken line. (**B**) Starvation-resistant cell lines (SW839, VMCR-RCW, and KMRC-1) were cultured in 25 mM or 0 mM glucose medium with or without 100 μM buformin for 24 h. The numbers of living and dead cells were counted using the trypan-blue exclusion assay. Error bars represent standard errors from three independent experiments. Asterisks (*) indicate statistically significant differences (p < 0.05) in respect of the numbers of living (upper) or dead cells (lower) under control conditions (25 mM glucose without buformin). Buformin inhibited mitochondrial oxidative phosphorylation in SW839 cells (**A**) with or without glucose and induced cell death for these cells (**B**) under glucose deprivation.

Buformin has previously been demonstrated to efficiently reduce cell survival under conditions of glucose deprivation for both cell types in RCC^10^. Therefore, we investigated whether buformin could also inhibit mitochondrial oxidative phosphorylation in starvation-resistant cells (Fig. 8). Similarly to etomoxir, buformin treatment (100 μM) significantly inhibited mitochondrial oxidative phosphorylation (Fig. 8A), and induced cell death in all starvation-resistant cells in the absence of glucose (Fig. 8B and Supplementary Table S5).

Interestingly, both etomoxir and buformin only had a significant cell death induction effect under conditions of no glucose (Figs 7C and 8B), even though both could inhibit mitochondrial oxidative phosphorylation with or without glucose (Figs 7A and 8A). These results suggest, although both agents target mitochondria, their effect is only harmful to cancer cells under the starvation conditions and are not toxic to normal cell lineages in the presence of nutrients. We propose that therapeutic inhibition of mitochondrial function will effectively induce cell death only in starvation-resistant cancer cells under the starvation conditions, and will induce a low level of side effects to normal cell lineages. Therefore, targeting mitochondrial function will offer a possible alternative therapeutic option to the presently used drugs for treatment-resistant RCC.

## Discussion

Here, we demonstrated that starvation-resistant cells in RCCs possessed higher mitochondrial oxidative phosphorylation activity than starvation-sensitive cells. The characteristics of both resistant and sensitive cell lines are summarized in Table 1. In starvation-resistant cells, increased mitochondrial performance was supported by the stored carbon sources, lipids and carbohydorates. Therefore, these cells resisted nutrient-deprivation cell death. The storage mechanism for lipid and glucose in starvation-resistant cells needs to be further investigated. Preliminary global transcriptional profiling has shown that *HMGCS1*, a lipid and cholesterol metabolism-related gene, and *SLC2A1/Glut1*, a glucose related gene, are up-regulated in starvation-resistant cells compared with starvation-sensitive cells (unpublished data). We will be able to confirm the differences in the two types through more comprehensive transcriptional profiling using a next generation sequencer. In clear cell RCCs, a major type of RCC, the cells contain abundant lipids and glycogen^14^. Clear cell RCCs generally carry genetic abnormalities of the VHL tumor suppresser genes. The starvation-resistant cell lines used in this study resembled clear cell RCCs, and were possibly associated with VHL gene mutations (Supplemental Table S6). However, VHL status in some of the starvation-sensitive cell lines is not controversially conclusive (Supplemental Table S6). Further investigation is in progress to clarify whether starvation-resistant cells are clear cell RCCs containing genetic abnormalities of VHL.

Starvation-resistant cells showed both lower mitochondrial ROS and higher SOD2 expression, and both characteristics can explain the differences in coupling efficiency, i.e. mitochondrial quality, between starvation-resistant and -sensitive cells. All the starvation-resistant cell lines had lower mitochondrial ROS due to higher SOD2 expression. Although Caki1 cells had a high level of SOD2 expression, they also had high mitochondrial ROS and were classified as a sensitive cell type that had lower mitochondrial activity, these cells showed induced cell death under glucose-deprivation conditions. Our data indicated that lower mitochondrial ROS and higher SOD2 expression essentially contribute to higher mitochondrial activity, a starvation-resistant cell phenotype.

We speculated that RCCs with poorest prognosis possessed higher mitochondrial function and high SOD2 activity, thus, SOD2 expression levels in primary lesions could be used as a predictive biomarker in prognosis. Indeed, patients with higher SOD2 expression in the primary lesions have been found to have significantly shorter survival periods than those with lower SOD2 expression; thus, a high level of SOD2 expression in the primary lesions could predict a worse prognosis in RCC. It has been reported that SOD2 expression levels increase with progression from early to late stage in many tumor types^15^. Patients, who showed higher SOD2 expression level in the RCC samples, clearly include cancer cells with higher SOD2 expression. Such cancer cells have the characteristics of high mitochondrial oxidative phosphorylation activity, similar to starvation-resistant cell lines, and can survive and expand under present therapeutic protocols. Therefore, patient survival is correlated with, and can be predicted by SOD2 expression level. One limitation of the present study is that the patient data came from a pilot study involving a limited number of clinical cases who had been treated by various targeting agents. We are now seeking to expand this study by investigating gene expression in RCC tissue samples using next generation sequencing. This should enable us to unambiguously identify the prognostic difference between starvation-sensitive and starvation-resistant cells in a clinical setting.

The present study suggested that the therapeutic inhibition of mitochondrial activity, which is supported by stored carbon sources and by low mitochondrial ROS via high SOD2 expression, could effectively target starvation-resistant cells in RCC. Etomoxir, an inhibitor of beta-oxidation, showed inhibition of mitochondrial activity in all starvation-resistant cell types (Fig. 7 and Supplementary Table S4), and induced cell death under starvation conditions. Similarly, buformin inhibited mitochondrial oxidative phosphorylation in all resistant cell lines (Fig. 8). This activity of buformin-induced cell death in resistant cell lines under glucose starvation was consistent with previous studies^16^,^17^. Recent reports^18^,^19^ have suggested that cancer cells which can survive under glucose deprivation within a tumor microenvironment possess higher mitochondrial oxidative phosphorylation activities, and that such cancer cells can circulate and form distant metastases. Therefore, high oxidative phosphorylation activity of cancer cells will become a potential therapeutic target for treating cancer patients. Buformin, a biguanide and potentially anti-tumorigenic agent that inhibits UPR^10^,^16^,^17^, reduced cell survival in both starvation-resistant and -sensitive RCC cell types under glucose deprivation conditions. Recently, it was reported that biguanides inhibit complex I and ATP synthase in mitochondrial oxidative phosphorylation^20^. In addition, both temsirolimus, an inhibitor of mTOR^21^, and azaserine, an inhibitor of GFPT1 in the hexosamine biosynthetic pathway^22^, did not induce cell death in starvation resistant cells^10^. Overall, the evidence indicates that therapeutic inhibition of mitochondrial activity will offer an effective option for RCCs with poor prognosis compared to the present therapies.

In conclusion, this study indicates that factors correlated with both the maintenance of high mitochondrial activity, such as SOD2 expression, and stores of lipids or carbohydrates are useful biomarkers and possible therapeutic targets in advanced malignancies such as metastatic and/or microenvironmentally starvation-resistant RCC.

## Methods

### Cell lines and cell culture conditions

We used seven RCC cell lines in this study, namely, Caki1, Caki2, NC65, ACHN, SW839, VMRC-RCW and KMRC-1. These cell lines were purchased from either the American Type Culture Collection, Riken Cell Bank, Cell Resource Center for Biomedical Research in Tohoku University or the Japanese Collection of Research Bioresources. All the cell lines were maintained in RPMI 1640 (Nakarai Tesque, Kyoto, Japan), which contained 25 mM glucose, supplemented with 10% fetal calf serum, penicillin (100 U/ml) and streptomycin (100 μg/ml) at 37 ^o^C in a humidified 5% CO_2_ atmosphere. In experimental cultures, cells were seeded in high-glucose medium and the culture medium was replaced on day 2 with fresh high-glucose medium (25 mM glucose) or with glucose deprivation medium (0 mM glucose, Nakarai Tesque). Cells on day 3 were used for some experiments. Treatments with etomoxir (500 μM) and buformin (100 μM) were carried out when the medium was replaced on day 2.

### Patients

The medical records of patients with RCC, who were treated in the Cancer Institute Hospital (Japanese Foundation for Cancer Research, Tokyo, Japan) between 2008 and 2014, were retrospectively reviewed. In all patients, clear cell RCC was confirmed by pathological diagnosis. Histopathology was reviewed according to the 2004 World Health Organization classification^23^. This study was carried out in compliance with the Helsinki declaration, and was approved by the institutional review board at Cancer Institute Hospital. Written informed consent was obtained from all of the subjects in this study.

### Measurement of oxygen consumption rate (OCR) and extracellular acidification rate (ECAR)

OCR and ECAR measurements were performed using an XF24^e^ Extracellular Flux analyzer (Seahorse Bioscience, North Billerica, MA) as previously descibed^13^. Briefly, cells were plated on XF24 cell culture plates (Seahorse Bioscience) and cultured for one day at 37 ^o^C in a humidified incubator with 5% CO_2_. Prior to the assay, the growth medium in the well of the XF cell plate was replaced with the appropriate assay medium. The sensor cartridge was calibrated, and the cell plate was incubated at 37 ^o^C for one hour. All experiments were performed at 37 ^o^C. After completion of the assay, DNA was extracted from the cells in each well using DNeasy Blood & Tissue Kit (Qiagen, Hilden, Germany); the extracted DNA was measured using a Smart Spec 3000 (Bio-Rad Laboratories, Hercules, CA) to assess cell number. The extracted DNA and subsequent qPCR were also used for counting mitochondrial numbers per each cell.

### Mitochondrial function assay

Mitochondrial function was assayed using an XF Cell Mito Stress Test Kit (Seahorse Bioscience) following the manufacturer’s instructions. Space respiratory capacity was calculated using the equation: ETC (electron transport chain) accelerator response/basal respiration. Coupling efficiency was calculated using the equation: 1- ATP coupler response/basal respiration. These calculations were similar to the representative OCR profile (Fig. 1A). The following compounds were injected: oligomycin (1 μM), an ATP synthetase inhibitor; FCCP, (0.5 μM), an uncoupler reagent; rotenone (1 μM), a mitochondrial complex I inhibitor; and antimycinA (1 μM), a mitochondrial complex III inhibitor.

### Glycolysis assay

The glycolysis assay was performed using an XF Glycolysis Stress Test Kit (Seahorse Bioscience) following the manufacturer’s instructions. Glycolysis and glycolytic capacity were calculated as for ECAR (Fig. 2A). The following compounds were injected: glucose (0.008–5 mM); oligomycin (1 μM), an ATP synthetase inhibitor; 2-deoxy-D-glucose (100 mM), glycolysis inhibitor.

### Mitochondrial function inhibition assay

The mitochondrial function inhibition assay was performed with etomoxir or buformin using an XF Cell Mito Stress Test Kit. Etomoxir (500 μM) was added to XF24 cell culture plates at 15 min before the start of the stress test. Buformin (100 μM) was added to XF24 cell culture plates one day before starting the stress test.

### Assay of lipids and carbohydrates in renal cell carcinomas

Observation and measurements of lipids in cells were performed by Oil Red O staining using a Lipid Assay kit (Cosmo Bio, Tokyo, Japan). Carbohydrates were observed by PAS staining^24^ and their levels were measured using the phenol-sulfuric acid method with a Total Carbohydrate Colorimetric Assay kit (BioVision, Milpitas, CA). Colorimetry of lipids and carbohydrates was carried out at 540 and 490 nm, respectively, using an InfinitM200 (TECAN, Mannendorf, Switzerland).

### Antibodies

The anti-SOD2 (#06–984) antibody was purchased from Millipore Corporation (Billerica, MA). The anti-α-tubulin (#T9026, DM1A) monoclonal antibody was purchased from Sigma-Aldrich (St. Louis, MO).

### Immunoblotting

Cells were lysed in Laemmli-SDS buffer, subjected to SDS-polyacrylamide gel electrophoresis, and electro-transferred to membrane filters (Immuno-Blot PVDF membranes, Bio-Rad Laboratories, Richmond, CA). The filters were incubated overnight with a primary antibody in TBS-T containing 2% bovine serum albumin and incubated for 1 hour in horseradish peroxidase-conjugated anti-mouse or anti-rabbit secondary antibody (Cell Signaling Technology) diluted 1:5,000 in TBS-T containing 2% BSA. Immunoreactivity was detected using the Luminata Classico Western HRP substrate (Millipore Corporation) with LAS4000 (Fujifilm, Tokyo, Japan) and quantified with MultiGauge software (Fujifilm), using an anti-α-tubulin antibody as the internal control.

### Detection of mitochondrial ROS

Cells (1.5 × 10^5^) were plated onto 6 well culture plates and cultured at 37 °C. MitoSoxRed was added to a final concentration of 5 μM for 30 min. The harvested cells were analyzed on an Attune^®^ Acoustic Focusing Cytometer (Life Technologies).

### Cell Viability

Cells (4 × 10^4^) were plated onto 24 well culture plates and cultured at 37 °C. For the cell viability assay, cells were stained with 0.1% trypan blue and the proportions of living and dead cells were determined.

### Quantitative reverse-transcription–polymerase chain reaction (qRT-PCR)

Total RNAs were obtained using acid guanidinium thiocyanate-phenol-chloroform^25^ from RCC cells and tissues from patients who had undergone radical nephrectomy. RCC tissues ware obtained from the primary lesions of patients who had given written informed consent. Quantitative RT-PCR was performed using the LightCycler 480 SYBG Master I Mix and LightCycler 480 System II (Roche Diagnostics, Mannheim, Germany). Gene expression was normalized against the *GAPDH* gene. Primer sequences are listed in Supplementary Table S7. All quantification analyses were performed in triplicate.

### Statistics

The data are reported as means ± S.E. The values were derived from at least three replicate experiments. Student’s *t*-test (two-tail) was used to compare differences between groups. In the clinical study, overall survival periods were measured from the initial administration of the targeted agents until death from any cause. Time-to-event distributions were estimated using Kaplan-Meier curves and statistically analyzed by the log-rank test. SPSS software was used for statistical analysis (SPSS for Windows, version 17.0, SPSS Inc.).

## Additional Information

**How to cite this article**: Isono, T. *et al.* Therapeutic inhibition of mitochondrial function induces cell death in starvation-resistant renal cell carcinomas. *Sci. Rep.* **6**, 25669; doi: 10.1038/srep25669 (2016).

## Supplementary Material

Supplementary Information

## Figures and Tables

**Table 1:** Summary of differences between resistant and sensitive RCC.

	Resistant RCC			Sensitive RCC			
	SW839	VMCR-RCW	KMRC-1	NC65	ACHN	Caki1	Caki2
Mitochondria quality							
Spare Respiratory Capacity (**under low glucose**)	+++	+++++	+++++	+	+	+	+
**Coupling Efficiency**	+++	+++	+++	+	+	+	+
Glycolytic Capacity (**under low glucose**)	+++	+++	+++	+	+	+	+
Carbon source							
**Carbohydrate content**	+++	+++++	+++++	+	+	+	+
Lipid content	+++	+++	+	+	+	+++	+++
Mitochondrial ROS (**under high glucose**)	+++	+	+++	+++++	+++	+++++	+++++
SOD2 expression							
qRT-PCR	+++	+++	+	+	+	+++++	+
Immunoblotting	+++	+++++	+	+	+	+++	+

The symbols, +++++ and +, indicate functions that are significantly higher (p < 0.05) and lower (p < 0.05), respectively, with respect to SW839 as the control function, indicates as +++. The marking (+++) in other cells can indicate no statistically significant difference from SW839. Bold letters indicate significant difference (p < 0.05) between the groups of resistant and sensitive RCC.
