# Supplementary material for: Therapeutic inhibition of mitochondrial function induces cell death in starvation-resistant renal cell carcinomas
Source: Sci Rep. 2016 May 9;6:25669. doi: 10.1038/srep25669 (PMC4860706; doi:10.1038/srep25669)
Supplement: Supplementary Information [file srep25669-s1.doc]

**Therapeutic inhibition of mitochondrial function induces cell death in starvation-resistant renal cell carcinomas.**

Takahiro Isono, Tokuhiro Chano, Junji Yonese and Takeshi Yuasa

**Supplementary Table**

**Table S1. OCR metrics for RCC. (DOC)**

**Table S2. The numbers of mitochondria per cell in RCC. (DOC)**

**Table S3. Characteristics of the metastatic RCC patients treated with targeting agents. (DOC)**

**Table S4. OCR metrics for RCC treated with Etomoxir. (DOC)**

**Table S5. OCR metrics for starvation-resistant RCC treated with Buformin. (DOC)**

**Table S6. *VHL* gene status of RCC. (DOC)**

**Table S7. Oligonucleotides used for qRT-PCR. (DOC)**

**Table S1. OCR Metrics for RCC.**

| **Cells__Glucose conc.** | **Basal respiration**  (pmol / min) | **/DNA**  (pmol / min / µg) | **Spare Respiratory Capacity** | **Coupling Efficiency** |
| --- | --- | --- | --- | --- |
| **Resistant-RCC**  SW839__25mM | 69.47 ± 1.42 | 112.05 | 1.52 ± 0.04 | 0.93 ± 0.03 |
| SW839__0mM | 61.14 ± 0.92 | 98.61 | 1.34 ± 0.02 | 0.88 ± 0.02 |
|  |  |  |  |  |
| VMCR-RCW__25mM | 108.79 ± 9.54 | 114.52 | 1.63 ± 0.17 | 0.86± 0.01 |
| VMCR-RCW__0mM | 94.80 ± 0.76 | 99.79 | 1.52 ± 0.02 | 0.87± 0.01 |
|  |  |  |  |  |
| KMRC-1__25mM | 93.09 ± 3.07 | 134.19 | 2.45 ± 0.07 | 0.92± 0.00 |
| KMRC-1__0mM | 88.45 ± 14.61 | 128.19 | 2.02 ± 0.04 | 0.87± 0.01 |
|  |  |  |  |  |
| **Sensitive-RCC** |  |  |  |  |
| NC65__25mM | 69.31 ± 1.37 | 33.16 | 1.07± 0.05 | 0.68 ± 0.01 |
| NC65__0mM | 78.58 ± 11.86 | 37.60 | 0.48 ± 0.01 | 0.63 ± 0.05 |
|  |  |  |  |  |
| ACHN__25mM | 85.37 ± 1.65 | 99.27 | 1.75± 0.05 | 0.55 ± 0.01 |
| ACHN__0mM | 125.22 ± 6.25 | 145.50 | 0.82 ± 0.02 | 0.48 ± 0.00 |
|  |  |  |  |  |
| Caki1__25mM | 126.96 ± 12.68 | 208.13 | 1.53± 0.09 | 0.66 ± 0.03 |
| Caki1__0mM | 55.35 ± 2.40 | 90.74 | 0.79 ± 0.03 | 0.60 ± 0.02 |
|  |  |  |  |  |
| Caki2__25mM  Caki2__0mM | 71.39 ± 3.06  61.71 ± 5.30 | 56.66  48.98 | 0.57± 0.02  0.30± 0.01 | 0.69 ± 0.04  0.49 ± 0.01 |

Basal respiration / DNA was normalized against each DNA content (µg).

**Table S2. The numbers of mitochondria per cell in RCC.**

|  | Mitochondria / Cell | | |  |
| --- | --- | --- | --- | --- |
|  | (*MT-ND* / *Alu*) | | |  |
| **Resistant-RCC** |  |  |  |  |
| SW839 | 1.00 | ± | 0.06 |  |
| VMCR-RCW | *2.05* | *±* | *0.09* |  |
| KMRC-1 | 0.95 | ± | 0.02 |  |
|  |  |  |  |  |
| **Sensitive-RCC** |  |  |  |  |
| NC65 | 1.4 | ± | 0.06 |  |
| ACHN | 2.7 | ± | 0.21 |  |
| Caki1 | *1.52* | *±* | *0.09* |  |
| Caki2 | 1.18 | ± | 0.07 |  |

The numbers of mitochondria per cell were calculated by qPCR using *MT-ND* from the mitochondrial genome and *Alu* from the nuclear genome. Quantitative PCR was performed using the LightCycler 480 SYBG Master I Mix and LightCycler 480 System II (Roche Diagnostics, Mannheim, Germany). All quantification analyses were performed in triplicate. The numbers were normalized with SW839 as control. Primer sequences are listed in Supplementary Table S6. The experimental data are reported as means ± S.E. Each mean represents data from independently triplicate experiments. Student’s *t* test (two-tail) was applied to compare differences between both groups. Italics indicate the statistically significant differences (p < 0.05) in respect to the value of SW839.

**Table S3 Characteristics of the metastatic RCC patients treated with targeting agents.**

|  | **SOD2 (High) (n=6)** | **SOD2 (Low) (n=10)** | **P value** |
| --- | --- | --- | --- |
| **Gender** |  |  |  |
| Male | 5 | 10 | 0.36 |
| Female | 1 | 0 |  |
| **Median Age (range)** | 67.5 (55-77) | 66 (51-75) | 0.75 |
| **Performance status** |  |  |  |
| 0 or 1 | 6 | 10 |  |
| 2 or greater | 0 | 0 |  |
| **Presence of metastasis** |  |  |  |
| **at diagnosis** |  |  |  |
| negative | 0 | 2 | 0.16 |
| positive | 6 | 8 |  |
| **Pathological diagnosis** |  |  |  |
| clear cell | 6 | 10 |  |
| non-clear cell | 0 | 0 |  |
| **Nephrectomy** |  |  |  |
| negative | 0 | 0 |  |
| positive | 6 | 10 |  |
| **Sarcomatoid componen**t | |  |  |
| negative | 3 | 9 | 0.15 |
| positive | 3 | 1 |  |
| **MSKCC classification** |  |  |  |
| Intermediate | 5 | 9 | 0.74 |
| Poor | 1 | 1 |  |

**Table S4. OCR Metrics for RCC treated with Etomoxir.**

| **Group__Glucose_Etomoxir** | **Basal**  **respiration** | | | | **ETC accelerator Response** | | | | **Coupling**  **Efficiency** | | |  |
| --- | --- | --- | --- | --- | --- | --- | --- | --- | --- | --- | --- | --- |
| **Resistant-RCC** |  |  |  |  |  |  |  |  |  |  |  | |
| SW839__25mM | 1.00 | ± | 0.02 |  | 1.66 | ± | 0.03 |  | 1.00 | ± | 0.02 | |
| SW839__0mM | 1.14 | ± | 0.02 |  | 1.39 | ± | 0.05 |  | 0.93 | ± | 0.01 | |
| SW839__25mM_Etomoxir | 0.05 | ± | 0.01 |  | 0.14 | ± | 0.01 |  | 1.12 | ± | 0.18 | |
| SW839__0mM_Etomoxir | 0.09 | ± | 0.02 |  | 0.19 | ± | 0.02 |  | 0.20 | ± | 0.04 | |
|  |  |  |  |  |  |  |  |  |  |  |  | |
| VMCR-RCW__25mM | 1.00 | ± | 0.02 |  | 1.59 | ± | 0.02 |  | 0.86 | ± | 0.01 | |
| VMCR-RCW__0mM | 0.88 | ± | 0.08 |  | 1.40 | ± | 0.03 |  | 0.86 | ± | 0.00 | |
| VMCR-RCW__25mM_Etomoxir | 0.11 | ± | 0.03 |  | 0.14 | ± | 0.03 |  | 0.09 | ± | 0.08 | |
| VMCR-RCW__0mM_Etomoxir | 0.11 | ± | 0.02 |  | 0.12 | ± | 0.01 |  | 0.18 | ± | 0.03 | |
|  |  |  |  |  |  |  |  |  |  |  |  | |
| KMRC-1__25mM | 1.00 | ± | 0.01 |  | 2.58 | ± | 0.04 |  | 0.88 | ± | 0.00 | |
| KMRC-1__0mM | 1.04 | ± | 0.03 |  | 1.73 | ± | 0.08 |  | 0.88 | ± | 0.00 | |
| KMRC-1__25mM__Etomoxir | 0.12 | ± | 0.01 |  | 0.16 | ± | 0.00 |  | 0.05 | ± | 0.08 | |
| KMRC-1__0mM__Etomoxir | 0.10 | ± | 0.01 |  | 0.18 | ± | 0.01 |  | -0.15 | ± | 0.09 | |
| **Sensitive-RCC** |  |  |  |  |  |  |  |  |  |  |  | |
| NC65__25mM | 1.00 | ± | 0.05 |  | 0.95 | ± | 0.05 |  | 0.68 | ± | 0.00 | |
| NC65__0mM | 0.96 | ± | 0.03 |  | 0.42 | ± | 0.02 |  | 0.59 | ± | 0.01 | |
| NC65__25mM_Etomoxir | 0.08 | ± | 0.01 |  | 0.07 | ± | 0.01 |  | 0.24 | ± | 0.09 | |
| NC65__0mM_Etomoxir | 0.12 | ± | 0.02 |  | 0.09 | ± | 0.01 |  | 0.52 | ± | 0.06 | |
|  |  |  |  |  |  |  |  |  |  |  |  | |
| Caki2__25mM | 1.00 | ± | 0.03 |  | 0.69 | ± | 0.02 |  | 0.70 | ± | 0.00 | |
| Caki2__0mM | 0.85 | ± | 0.08 |  | 0.24 | ± | 0.02 |  | 0.52 | ± | 0.01 | |
| Caki2__25mM_Etomoxir | 0.10 | ± | 0.01 |  | 0.05 | ± | 0.01 |  | 0.40 | ± | 0.07 | |
| Caki2__0mM_Etomoxir | 0.30 | ± | 0.02 |  | 0.18 | ± | 0.01 |  | 0.31 | ± | 0.03 | |
|  |  |  |  |  |  |  |  |  |  |  |  | |

Basal respiration and ETC accelerator responses were normalized against 25mM glucose.

**Table S5. OCR Metrics for starvation-resistant RCC treated with Buformin.**

| **Group_ Glucose_ Buformin** | **Basal**  **Respiration** | | | | **ETC accelerator Response** | | | | **Coupling**  **Efficiency** | | |  |
| --- | --- | --- | --- | --- | --- | --- | --- | --- | --- | --- | --- | --- |
|  |  |  |  |  |  |  |  |  |  |  |  | |
| SW839__25mM | 1.00 | ± | 0.13 |  | 1.73 | ± | 0.08 |  | 0.94 | ± | 0.02 | |
| SW839__0mM | 1.06 | ± | 0.02 |  | 1.57 | ± | 0.09 |  | 0.88 | ± | 0.02 | |
| SW839__25mM_Buformin | -0.17 | ± | 0.02 |  | 0.29 | ± | 0.04 |  | 0.19 | ± | 0.07 | |
| SW839__0mM_Buformin | -0.08 | ± | 0.02 |  | 0.35 | ± | 0.03 |  | -0.38 | ± | 0.45 | |
|  |  |  |  |  |  |  |  |  |  |  |  | |
| VMCR-RCW__25mM | 1.00 | ± | 0.02 |  | 1.45 | ± | 0.05 |  | 0.82 | ± | 0.01 | |
| VMCR-RCW__0mM | 0.90 | ± | 0.02 |  | 1.39 | ± | 0.02 |  | 0.85 | ± | 0.01 | |
| VMCR-RCW__25mM_Buformin | 0.03 | ± | 0.02 |  | 0.13 | ± | 0.01 |  | 0.34 | ± | 0.27 | |
| VMCR-RCW__0mM_Buformin | 0.11 | ± | 0.04 |  | 0.16 | ± | 0.02 |  | 0.23 | ± | 0.07 | |
|  |  |  |  |  |  |  |  |  |  |  |  | |
| KMRC-1__25mM | 1.00 | ± | 0.06 |  | 2.66 | ± | 0.19 |  | 0.92 | ± | 0.02 | |
| KMRC-1__0mM | 1.21 | ± | 0.03 |  | 2.46 | ± | 0.09 |  | 0.88 | ± | 0.02 | |
| KMRC-1__25mM_Buformin | 0.11 | ± | 0.03 |  | 0.21 | ± | 0.01 |  | 0.40 | ± | 0.02 | |
| KMRC-1__0mM_Buformin | 0.03 | ± | 0.07 |  | 0.29 | ± | 0.03 |  | 0.37 | ± | 0.45 | |
|  |  |  |  |  |  |  |  |  |  |  |  | |

Basal respiration and ETC accelerator responses were normalized against 25mM glucose.

**Table S6. *VHL* gene status of RCC.**

|  | ***VHL* gene status** | |  |
| --- | --- | --- | --- |
| **Resistant-RCC** |  |  | |
| SW839 | Mutant* |  | |
| VMCR-RCW | No data |  | |
| KMRC-1 | Mutant† |  | |
|  |  |  | |
| **Sensitive-RCC** |  |  | |
| NC65 | No data |  | |
| ACHN | Wild* / Mutant‡ |  | |
| Caki1 | Wild* |  | |
| Caki2 | Wild* / Mutant‡ |  | |

*: Shinojima T. *et al.*; Renal cancer cells lacking hypoxia inducible factor (HIF)-1a expression maintain vascular endothelial growth factor expression through HIF-2a. *Carcinogenesis.* **28**, 529-536 (2007).

†: Data sheet from Tohoku University or the Japanese Collection of Research Bioresources.

‡: Whaley J. M.*et al.*; Germ-line mutation in the von Hippel-Lindau tumor-suppressor gene are similar to somatic von Hippel-Lindau aberrations in sporadic renal cell carcinoma. *Am. J. Hum. Genet.* **55**, 1092-1102 (1994).

**Table S7. Oligonucleotides used for qRT-PCR**

| Gene | Forward (5’ to 3’) | Reverse (5’ to 3’) |
| --- | --- | --- |
| *SOD2*  *GAPDH*  *MT-ND1*  *Alu* | CTGCACCACAGCAAGCACCAC  GGGAGCCAAAAGGGTCATCATC  CCCTAAAACCCGCCACATCT  CTTGCAGTGAGCCGAGATT | ACCGTTAGGGCTGAGGTTTGTC  TGGCATGGACTGTGGTCATGAG  GAGCGATGGTGAGAGCTAAGGT  GAGACGGAGTCTCGCTCTGTC |
